# Supplementary material for: The Helicobacter pylori methylome is acid-responsive due to regulation by the two-component system ArsRS and the type I DNA methyltransferase HsdM1 (HP0463)
Source: J Bacteriol. 2024 Jan 5;206(1):e00309-23. doi: 10.1128/jb.00309-23 (PMC10810217; doi:10.1128/jb.00309-23)
Supplement: Supplemental legends — Legends for supplemental figures and tables. [file jb.00309-23-s0003.docx]

**Supplemental Material**

**Table S1.** ***H. pylori* 26695 Type I Restriction-Modification Systems.**

**Figure S1. Neither *hsdM*2 (HP0850) nor *hsdM*3 (HP1403), the other Type I DNA Methyltransferases in *H. pylori* 26695, exhibit ArsS-dependent or acid-sensitive transcription.** **A,C)** *Both the control H. pylori mutant, possessing an intact arsRS locus, and an isogenic ΔarsS mutant were grown to mid-logarithmic stage of growth and equal aliquots then harvested. mRNA levels are expressed as relative quantities in relation to the control mutant.* ***B,D)*** *After growth of the control H. pylori mutant and isogenic ΔarsS mutant to mid-logarithmic phase, equal aliquots were harvested and resuspended in pH 7 or pH 5 broth. mRNA levels are expressed as relative quantities in relation to the time zero sample.* ***A)*** *The expression of hsdM2 in the H. pylori 26695 control and ΔarsS mutants.* ***B)*** *The expression of hsdM2 in the H. pylori 26695 control and ΔarsS mutants at pH 7 or pH 5 at 30 and 60 minutes.* ***C)*** *The expression of hsdM3 in the H. pylori 26695 control and ΔarsS mutants.* ***D)*** *The expression of hsdM3 in the H. pylori 26695 control and ΔarsS mutants at pH 7 or pH 5 at 30 and 60 minutes. This experiment was performed three times, yielding three distinct biological replicates, each represented by a dot. Error bars; standard deviation.*

**Figure S2. Neither** ***M.HpyAI* (HP1208) nor *M.HpyAII* (HP1368), the Type II DNA Methyltransferases used as controls in *H. pylori* 26695, exhibit ArsS-dependent or acid-sensitive transcription.** **A,C)** *Both the control H. pylori mutant, possessing an intact arsRS locus, and an isogenic ΔarsS mutant were grown to mid-logarithmic stage of growth and equal aliquots then harvested. mRNA levels are expressed as relative quantities in relation to the control mutant.* ***B,D)*** *After growth of the control H. pylori mutant and isogenic ΔarsS mutant to mid-logarithmic phase, equal aliquots were harvested and resuspended in pH 7 or pH 5 broth. mRNA levels are expressed as relative quantities in relation to the time zero sample.* ***A)*** *The expression of M.HpyAI in the H. pylori 26695 control and ΔarsS mutants.* ***B)*** *The expression of M.HpyAI in the H. pylori 26695 control and ΔarsS mutants at pH 7 or pH 5 at 30 and 60 minutes.* ***C)*** *The expression of M.HpyAII in the H. pylori 26695 control and ΔarsS mutants.* ***D)*** *The expression of M.HpyAII in the H. pylori 26695 control and ΔarsS mutants at pH 7 or pH 5 at 30 and 60 minutes. This experiment was performed three times, yielding three distinct biological replicates, each represented by a dot. Error bars; standard deviation.*

**Table S2.** **ThermoFisher TaqMan assay sequences used for qRT-PCR probes.** *The reporters have FAM as the fluorophore at the 5’ end and NQR as the quencher at the 3’ end.*
