# Supplementary material for: The Helicobacter pylori methylome is acid-responsive due to regulation by the two-component system ArsRS and the type I DNA methyltransferase HsdM1 (HP0463)
Source: J Bacteriol. 2024 Jan 5;206(1):e00309-23. doi: 10.1128/jb.00309-23 (PMC10810217; doi:10.1128/jb.00309-23)
Supplement: Table S1 — H. pylori 26695 Type I restriction-modification systems. [file jb.00309-23-s0004.docx]

| **Locus Tag** | **Gene Name** | **Protein Type** |
| --- | --- | --- |
| HP0464 | *hsdR*1 | Restriction Endonuclease |
| HP0463 | *hsdM*1 | DNA Methyltransferase |
| HP0462 | *hsdS*1b | Specificity Subunit |
| HP0846 | *hsdR*2 | Restriction Endonuclease |
| HP0850 | *hsdM*2 | DNA Methyltransferase |
| HP0848 | *hsdS*2 | Specificity Subunit |
| HP1402 | *hsdR*3 | Restriction Endonuclease |
| HP1403 | *hsdM*3 | DNA Methyltransferase |
| HP1404 | *hsdS*3b | Specificity Subunit |
| HP0790 | *hsdS*5 | Specificity Subunit |
| HP1383 | *hsdS*6 | Specificity Subunit |
