# Supplementary material for: The Helicobacter pylori methylome is acid-responsive due to regulation by the two-component system ArsRS and the type I DNA methyltransferase HsdM1 (HP0463)
Source: J Bacteriol. 2024 Jan 5;206(1):e00309-23. doi: 10.1128/jb.00309-23 (PMC10810217; doi:10.1128/jb.00309-23)
Supplement: Table S2 — ThermoFisher TaqMan assay sequences used for qRT-PCR probes. [file jb.00309-23-s0005.docx]

| **Target** | **Forward Probe (5’-3’)** | **Reverse Probe (3’-5’)** | **Reporter (5’-3’)** |
| --- | --- | --- | --- |
| GyrB - DNA Gyrase B subunit (consensus normalizing gene) (*gyrB*) | AAAGCCAGAGAGCTTACAAGGAAAA | CGCCCTCCACTAAAAAGATTTCACT | TTGCCTGGAAAATTAG |
| HP0463- Type I DNA methyltransferase enzyme (*hsdM*1) | ATGAGCCGACTAGAAATGTCAAAATCT | CCTATTTGGTGGGCTAATGCCATT | CCTGTGCCTGCACTTG |
| HP0850 - Type I DNA methyltransferase enzyme (*hsdM*2) | AAAGTGTTAGGCGATAAAAATGTCTCAAAAG | GGCAAAGGTTGTAAGTGGTCAAATT | TCTTGCCCAAAATACC |
| HP1403 - Type I DNA methyltransferase enzyme (*hsdM*3) | CGCGCGCCAGAAAGG | AGCGGTTTTTATTGCCGTCTTTTT | CCTTGCTCGCATCTAT |
| HP1208 - Type II DNA methyltransferase enzyme (*M.HpyAI*) | GCCTTAAAAAAAGCGCTCAAAAAGA | TGCTGATACACTTCGTTAGCGTTTA | AAAGGCGCTGATTTTG |
| HP1368 - Type IIS DNA methyltransferase enzyme (*M.HpyAII*) | ACATGCTAAAAAACAAACCTAAAATGTTCTTACT | GGGTAGCTCCCAAACATCAATCTTT | ACGCGCAAATCCCAC |
